# A Conceptual Wound-Oriented Reinterpretation of Perfusion Heterogeneity in Chronic Limb-Threatening Ischemia

**DOI:** 10.3390/jcm15114119

**Published:** 2026-05-26

**Authors:** Mircea Ionut Popitiu, Lorenzo Patrone, Giacomo Clerici, Serban Comsa, Gloria Gavrila-Ardelean, Nilima Rajpal Kundnani, Nicu Olariu, Mihai Edmond Ionac

**Affiliations:** 1 Research Center in Vascular and Endovascular Surgery, “Victor Babes” University of Medicine and Pharmacy, 300041 Timisoara, Romania; mihai.ionac@umft.ro; 2 Vascular and Endovascular Surgery Unit, San Giovanni di Dio Hospital, 50143 Florence, Italy; patropatronis@gmail.com; 3 San Carlo Clinic, 20026 Paderno Dugnano, Italy; gclerici@casacura.it; 4 Angiogenesis Research Center, Department II Morphologic Microscopy/Histology, “Victor Babes” University of Medicine and Pharmacy, 300041 Timisoara, Romania; serban.comsa@umft.ro; 5 Faculty of Educational Sciences, Psychology and Social Work, “Aurel Vlaicu” University of Arad, 310032 Arad, Romania; 6 University Clinic of Internal Medicine and Ambulatory Care, Prevention and Cardiovascular Recovery, Department VI-Cardiology, “Victor Babes” University of Medicine and Pharmacy, 300041 Timisoara, Romania; knilima@umft.ro; 7 Research Center of Timisoara, Institute of Cardiovascular Diseases, “Victor Babes” University of Medicine and Pharmacy, 300041 Timisoara, Romania; 8 Division of Nephrology, Department of Internal Medicine II, “Victor Babes” University of Medicine and Pharmacy, 300041 Timisoara, Romania; nicu.olariu@umft.ro; 9 Center for Molecular Research in Nephrology and Vascular Disease, “Victor Babes” University of Medicine and Pharmacy, 300041 Timisoara, Romania; 10 Doctoral School, “Victor Babes” University of Medicine and Pharmacy, 300041 Timisoara, Romania

**Keywords:** chronic limb-threatening ischemia, angiosome, woundosome, infrapopliteal revascularization, wound healing, limb salvage, perfusion, collateral circulation, microvascular function, endovascular therapy

## Abstract

**Background/Objectives:** The angiosome concept is widely used to guide infrapopliteal revascularization in patients with chronic limb-threatening ischemia (CLTI). However, clinical outcomes are not always fully explained by anatomical target-artery alignment alone. The present study aimed to revisit a previously published angiosome-based cohort through a wound-oriented conceptual perspective and to explore whether perfusion heterogeneity may help contextualize variability in clinical outcomes. **Methods:** This retrospective secondary analysis included 51 patients with CLTI who underwent infrapopliteal endovascular revascularization. Patients were originally classified as direct, indirect, or mixed revascularization according to angiosome-based criteria. The present study represents an exploratory conceptual reinterpretation of the original dataset. No new variables were introduced, and the woundosome concept was not operationalized as a measurable patient-level variable. Statistical analyses were exploratory and descriptive in nature. **Results:** High rates of wound healing and limb salvage were observed across all revascularization patterns at 12 months. Within the limitations of this small exploratory cohort, no consistent detectable differences in clinical outcomes were observed across anatomical revascularization patterns. Stratification according to the number of affected angiosomes did not reveal clear outcome differences. The findings suggest that factors beyond anatomical target-artery alignment may contribute to wound healing variability in CLTI. **Conclusions:** The present study does not establish a validated wound-oriented perfusion model but highlights the limitations of relying exclusively on anatomical angiosome classification when interpreting clinical outcomes in CLTI. In this context, the woundosome may serve as a descriptive and hypothesis-generating conceptual framework for discussing perfusion heterogeneity and wound-level perfusion complexity. Prospective studies integrating objective perfusion assessment and standardized wound evaluation are required.

## 1. Introduction

As the end-stage manifestation of peripheral arterial disease, chronic limb-threatening ischemia (CLTI) carries significant risks, including limb loss, high morbidity, and increased mortality [1]. For patients with tissue loss, restoring sufficient perfusion at the wound site is essential in order to achieve wound healing and limb salvage [2]. Infrapopliteal endovascular revascularization has therefore become a cornerstone of treatment in this population [3,4].

The angiosome concept, which was originally introduced by Taylor and Palmer, provides an anatomical framework in which the foot and ankle are divided into separate vascular regions, each supplied by a specific source artery [5]. This model has been widely adopted for guiding revascularization strategies, as it supports direct revascularization through the artery that supplies the angiosome which corresponds to the wound [6]. Although multiple studies and meta-analyses suggest that angiosome-guided interventions result in improved outcomes, the evidence remains inconsistent, especially in patients characterized by complex disease, multi-territory wounds or extensive collateral circulation [7,8,9].

Increasing clinical evidence indicates that clinical outcomes in CLTI patients may not be fully accounted for solely by anatomical target-artery alignment [4]. In addition to macroscopic arterial inflow, wound healing also depends on the integrity of collateral circulation, microvascular function and individual anatomical variability [10,11]. In the case of advanced disease, perfusion to the wound bed can be maintained through alternative vascular pathways, which extend beyond the predefined angiosome boundaries [10,11,12,13].

In the previously published angiosome-based analysis by Popitiu et al., favorable outcomes were observed following direct revascularization, although outcome differences were not consistently statistically significant and favorable evolution was also observed in selected indirect revascularization cases [7]. These findings suggest that the complexity of tissue perfusion in CLTI may not be reflected adequately by a classification that is purely anatomical.

In this context, a more functional, wound-oriented interpretation of perfusion may be required. The concept of the woundosome has been proposed to describe the patient-specific perfusion territory that effectively supplies the wound [14]. Unlike the angiosome, which is anatomically defined, the woundosome represents a dynamic and functional unit integrating direct arterial inflow, collateral circulation, and multi-vessel contributions [14,15,16].

The aim of the present study was to revisit a previously published angiosome-based cohort from a wound-oriented conceptual perspective and to explore whether perfusion heterogeneity may help contextualize variability in wound healing and limb salvage beyond a purely anatomical direct-versus-indirect classification framework [7,14,15,16].

Importantly, the present manuscript was not designed as a validation study of the woundosome concept. Rather, it represents an exploratory conceptual reinterpretation of a previously published dataset intended to discuss the potential limitations of relying exclusively on anatomical angiosome classification when interpreting clinical outcomes in CLTI [14,15,16]. No additional patients, interventions, or outcome data were included.

For clarity, the principal methodological and conceptual differences between the previously published study and the present exploratory reanalysis are summarized in Table 1.

## 2. Materials and Methods

### 2.1. Study Design and Population

This study is a retrospective secondary analysis of a previously published single-center cohort comprising 51 patients with CLTI who were treated using infrapopliteal endovascular revascularization. No new data were collected, and the original dataset was reanalyzed without modification.

The analytical workflow of the present study focused on the exploratory interpretation of perfusion patterns and their relationship with clinical evolution following infrapopliteal revascularization.

In the original analysis, 28 patients underwent direct revascularization, 12 indirect revascularization, and 11 were classified as having mixed revascularization patterns. The same cohort was retained in the present study without changes in patient selection or data structure.

Mixed revascularization patterns were preserved as a distinct subgroup, as these cases likely reflect complex perfusion scenarios not adequately captured by a binary direct-versus-indirect classification.

### 2.2. Revascularization Strategy (Original Classification)

The original analysis classified revascularization as direct when the target artery that supplied the angiosome corresponding to the wound location was successfully revascularized and indirect when revascularization was achieved through a different artery with presumed perfusion via collateral circulation.

However, this definition may not fully align with the original and subsequently expanded angiosome concept as described by Taylor and Palmer, which incorporates both anatomical and functional angiosomes [5]. In this broader interpretation, pedal arterial arches and collateral networks are considered integral components of intentional topographic revascularization [5,16]. Consequently, perfusion achieved through these pathways may, in certain contexts, be regarded as functionally consistent with direct revascularization, and a strict binary classification may lead to potential misclassification of revascularization strategies.

This angiosome-based classification was preserved in the present study and used for comparative analyses. This classification reflects the anatomical framework that was applied in the original dataset and is not derived from the woundosome concept.

Angiographic classification and interpretation were based on the original procedural assessments performed by the treating vascular specialists during the initial study and were derived from the consensus procedural documentation available in the dataset. No formal interobserver agreement analysis was performed in the present retrospective reanalysis.

### 2.3. Wound-Oriented Perfusion Framework

For the present reanalysis, a wound-oriented conceptual framework was used to provide a descriptive interpretation of perfusion heterogeneity in relation to clinical outcomes [14,15,16]. This framework was not intended as a measurable physiological model or as a validated clinical classification system. Instead, it was used as a conceptual approach to discuss the possible interaction between direct arterial inflow, collateral circulation, multivessel perfusion, and local wound-level perfusion complexity [10,11,12,13,14,15,16].

Importantly, the woundosome concept was not operationalized as a patient-level variable within the present dataset and was therefore not evaluated as an independent predictor of wound healing or limb salvage. In addition, no standardized wound perfusion scoring system, microcirculatory assessment, or functional perfusion measurements were available for retrospective analysis [12,14,15].

The interpretive approach was based primarily on angiographic and procedural findings available in the original cohort dataset. However, direct physiological assessment of tissue perfusion, including transcutaneous oxygen pressure, skin perfusion pressure, or other microvascular functional measurements, was not available [12,17,18]. Consequently, the present analysis should be regarded as descriptive and hypothesis-generating rather than quantitative or clinically validated.

#### Analytical Handling of Mixed Revascularization Patterns

Cases classified as mixed revascularization were retained as a distinct subgroup because of their heterogeneous perfusion patterns that reflect combined or non-standard revascularization pathways. These cases likely represent complex clinical scenarios that are not adequately captured by a simplified binary classification framework. Therefore, they were included in descriptive analyses but excluded from direct versus indirect inferential comparisons. This decision was made to preserve internal consistency within the binary comparisons, as mixed cases could not be reliably assigned to a single perfusion category without introducing classification bias.

### 2.4. Data Collection and Variables

Clinical, procedural and outcome data were derived from the original dataset without the introduction of new measured variables. The following categories of variables were analyzed:

- Demographic data (age, sex);
- Cardiovascular risk factors (diabetes mellitus [oral and insulin-treated], hypertension, dyslipidemia, smoking);
- Comorbidities (renal insufficiency, hemodialysis, coronary artery disease, heart failure, cerebrovascular disease);
- Lesion and wound characteristics (wound location [anterior tibial, posterior tibial, peroneal], multi-territorial involvement, number of affected angiosomes);
- Procedural variables (number of angioplasties, target vessels treated, stent implantation); and
- Clinical outcomes (wound healing at 12 months, limb salvage at 12 months, minor and major amputation, and mortality).

It should be noted that several clinically relevant local factors known to influence wound healing were not available in the dataset. These include the presence of local infection or sepsis, the extent of tissue necrosis, peripheral neuropathy, microangiopathy, and venous insufficiency. The absence of these variables limits the ability to fully account for wound-specific determinants of healing and may partially explain discrepancies between anatomical or procedural findings and clinical outcomes.

In addition to the original variables, surrogate markers of perfusion complexity were considered, including the number of treated infrapopliteal vessels, the number of affected angiosomes and the presence of multi-territorial wounds. These variables were used as indirect indicators of perfusion complexity and were explored in relation to clinical outcomes through descriptive and correlation-based analyses. However, they do not represent direct measures of tissue perfusion or oxygenation and should be interpreted accordingly.

### 2.5. Outcomes

The primary outcomes of interest were wound healing at 12 months and limb salvage at 12 months, as recorded in the original dataset. Wound healing was considered achieved when the index wound was completely epithelialized. Limb salvage was defied as the avoidance of major (above-ankle) amputation during follow-up.

Secondary outcomes included minor amputation, major amputation and all-cause mortality. All outcomes were assessed based on the available clinical follow-up data from the original cohort.

### 2.6. Statistical Analysis

Statistical analyses were performed using IBM SPSS Statistics for Windows, version 27.0 (IBM Corp., Armonk, NY, USA).

The statistical analysis was primarily exploratory, reflecting the retrospective design and relatively small sample size of the study. No formal sample size or power calculation was performed, as the analysis was based on a fixed, previously published dataset.

Continuous variables were assessed for normality using the Shapiro–Wilk test and are presented as mean ± standard deviation or median (interquartile range), as appropriate. Categorical variables are presented as frequencies and percentages.

Comparisons between the direct and indirect revascularization groups were performed using Student’s *t*-test or the Mann–Whitney U test for continuous variables, depending on data distribution. Categorical variables were compared using the chi-square test or Fisher’s exact test, as appropriate. Mixed revascularization cases were excluded from inferential comparisons, as described previously, due to their heterogeneous perfusion characteristics and the inability to reliably assign them to a single perfusion category.

Spearman’s rank correlation coefficient was used to explore potential associations between selected clinical variables and clinical outcomes, including wound healing and limb salvage.

Given the limited sample size and low number of outcome events, multivariable regression modeling was considered methodologically unreliable and was therefore not performed. The available sample size and event distribution were insufficient for reliable multivariable outcome modeling.

Due to missing data within the original dataset, analyses were performed using available-case data; therefore, the number of evaluable cases varied across analyses.

All statistical tests were two-sided, and a *p*-value < 0.05 was considered statistically significant. However, given the exploratory nature of the study, the results were interpreted cautiously, with greater emphasis placed on descriptive trends and overall patterns rather than on statistical significance alone.

### 2.7. Methodological Considerations

There are several methodological considerations that should be acknowledged. The present study represents a retrospective reinterpretation of a previously published dataset derived from a relatively small single-center cohort, which limits the robustness of the analyses and the broader applicability of the findings. Although WIfI-related information was partially available from the original cohort, these data were not consistently documented and could not be reliably integrated into the present reanalysis. This is an important limitation considering the wound-oriented focus of the study.

Another limitation is related to the exclusion of mixed revascularization cases from the binary comparative analyses. These cases reflect more complex perfusion patterns that are difficult to classify within a simplified direct-versus-indirect framework and may have introduced selection bias.

In addition, the interpretation of the findings is limited by the absence of objective perfusion measurements. The analysis was based mainly on angiographic and procedural data and did not include a structured evaluation of local wound-related factors such as infection, extent of tissue loss, neuropathy, or microvascular dysfunction. Therefore, the relationship between arterial flow restoration and actual wound healing cannot be fully characterized within the present study.

The proposed wound-oriented interpretation should be regarded as exploratory and requires further evaluation in prospective clinical studies.

### 2.8. Ethical Considerations

This study represents a retrospective reanalysis of a previously published dataset. The original study was conducted in accordance with the principles of the Declaration of Helsinki and received approval from the relevant institutional ethics committees.

The present analysis was based exclusively on previously collected and fully anonymized data. No new data were collected, and no patient-identifiable information was accessed.

In accordance with institutional policies and applicable regulations, additional ethical approval and informed consent were not required for this secondary analysis of anonymized data, and the requirement for ethical approval was therefore waived.

## 3. Results

### 3.1. Patient Characteristics

The analysis included a total of 51 patients with CLTI. According to the original angiosome-based classification, 28 patients underwent direct revascularization, 12 indirect revascularization, and 11 demonstrated mixed revascularization patterns.

In the available-case analysis, baseline demographic and clinical characteristics of the patients were broadly comparable between the direct and indirect revascularization groups. Cardiovascular risk factors were highly prevalent in the cohort, including diabetes mellitus and hypertension, with a substantial comorbidity burden. Renal dysfunction, including patients requiring hemodialysis, was also frequently observed, reflecting the complexity of a typical CLTI population.

### 3.2. Procedural Characteristics

Infrapopliteal angioplasty was performed targeting the anterior tibial, posterior tibial, or peroneal arteries, either as single-vessel or multi-vessel interventions.

Direct revascularization was defined as restoration of blood flow to the artery supplying the angiosome corresponding to the wound location, whereas indirect revascularization relied on collateral perfusion from adjacent vascular territories. Mixed revascularization patterns involved multi-vessel interventions or combined perfusion pathways.

The direct and indirect revascularization groups had comparable baseline demographic and clinical characteristics, with no statistically significant differences observed across most variables, suggesting a broadly similar baseline risk profile.

Indirect and mixed revascularization strategies were more frequently associated with multi-vessel interventions and a greater number of treated infrapopliteal arteries, suggesting increased procedural complexity and reflecting more advanced or diffuse disease patterns. These observations were descriptive and were not formally tested.

Although WIfI-related data had been reported in the previous publication, these data were not systematically incorporated into the present exploratory reanalysis. In addition, no structured clinical assessment of wound characteristics or microcirculatory status was incorporated into the current analytical framework. Consequently, the interpretation of revascularization strategies remains primarily based on angiographic and procedural findings, without direct correlation to the clinical heterogeneity of tissue loss or local factors influencing wound healing.

### 3.3. Clinical Outcomes

Comparative analyses were performed at the patient level between direct and indirect revascularization groups. Mixed revascularization cases were not included in these binary comparisons due to their heterogeneous perfusion characteristics and the inability to reliably assign them to a single category without introducing classification bias.

Clinical outcomes were analyzed descriptively across the direct, indirect, and mixed revascularization groups. At 12 months, wound healing and limb salvage were achieved in a substantial proportion of patients across all groups, consistent with the outcomes reported in the original analysis. No statistically significant differences were observed between direct and indirect revascularization strategies.

Favorable clinical outcomes were also observed in some patients who underwent indirect revascularization despite the absence of direct target-artery alignment. However, given the limited size of the indirect revascularization subgroup, these findings should be interpreted cautiously and not as evidence of equivalence between revascularization strategies.

The mixed revascularization subgroup showed heterogeneous outcomes, likely reflecting complex perfusion patterns involving both direct arterial inflow and collateral circulation.

**Figure 1:**
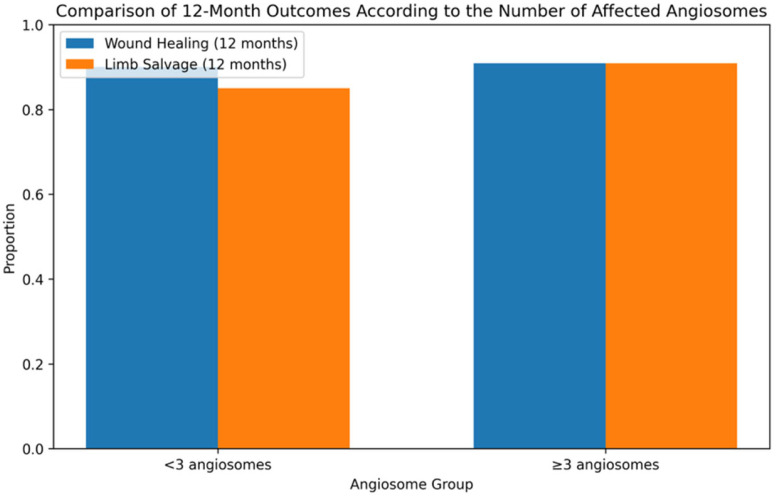
Comparison of 12-month wound healing and limb salvage rates according to the number of affected angiosomes (<3 vs. ≥3).

To further explore the role of anatomical perfusion complexity, patients were stratified according to the number of affected angiosomes (<3 vs. ≥3). No significant differences between the two groups were observed in 12-month wound healing or limb salvage, as shown in Figure 1.

Healing rates were 90% in patients with <3 affected angiosomes and 91% in those with ≥3 affected angiosomes. Limb salvage rates were 85% and 91%. However, these findings should be interpreted cautiously given the limited sample size and exploratory nature of the analysis (Fisher’s exact test, *p* = 1.0 for both comparisons).

Table 2 presents a comparison of clinical outcomes between the direct and indirect revascularization groups.

Overall, these findings indicate that anatomical target-artery alignment and the extent of angiosomal involvement alone may not fully account for clinical outcomes in this cohort.

The number of available cases varied across analyses depending on data completeness, and all results are presented based on available-case datasets. Differences in sample size therefore reflect missing data within the original dataset rather than cohort expansion.

**Figure 2:**
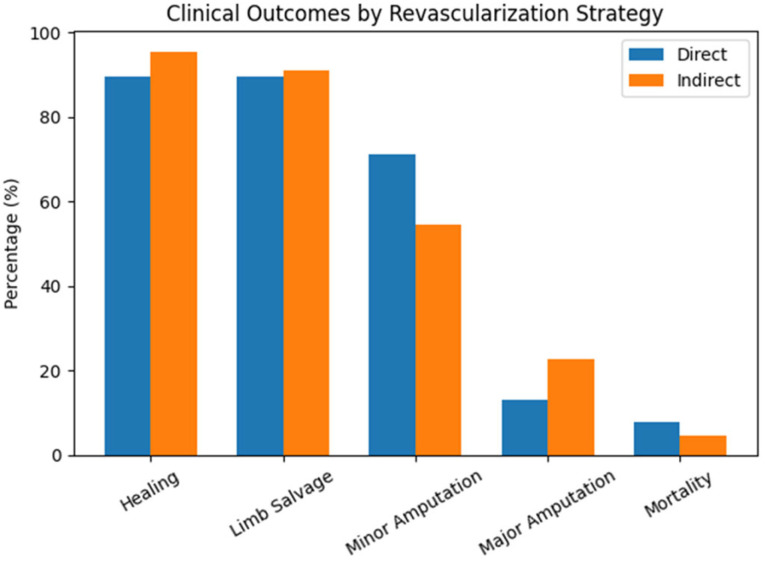
Clinical outcomes at 12 months according to direct and indirect revascularization strategies.

Clinical outcomes according to revascularization strategy are illustrated in Figure 2, showing comparable rates of wound healing and limb salvage between direct and indirect revascularization groups.

**Figure 3:**
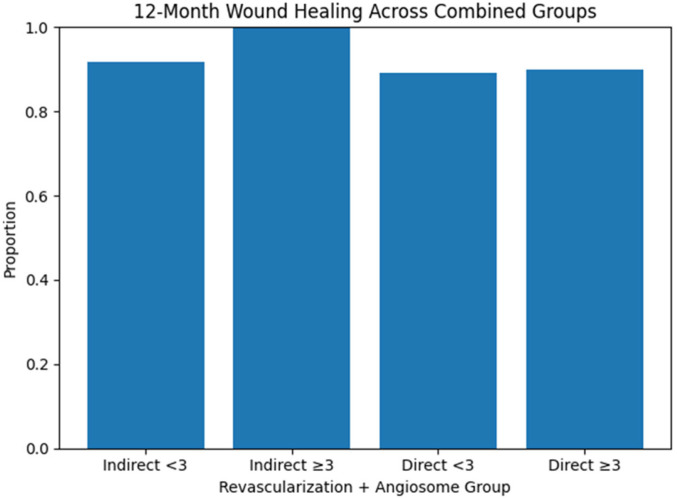
Twelve-month wound healing rates according to combined stratification by revascularization type (direct vs. indirect) and number of affected angiosomes (<3 vs. ≥3).

Figure 3 presents the combined stratification analysis.

### 3.4. Correlation Analysis

Exploratory Spearman correlation analysis was performed using available-case data within the direct and indirect revascularization subgroups. Spearman correlation coefficients were calculated for different clinical outcomes (wound healing and limb salvage). Selected variables included the number of affected angiosomes, renal dysfunction, hemodialysis and type of revascularization.

No statistically significant correlations were found between clinical outcomes and the analyzed variables. The number of affected angiosomes showed no clear association with wound healing or limb salvage within the limitations of this exploratory cohort (wound healing: r = −0.15, *p* = 0.37; limb salvage: r = 0.09, *p* = 0.61). Similarly, renal dysfunction and hemodialysis status were not significantly correlated with outcomes, and the type of revascularization (direct versus indirect) was not associated with wound healing or limb salvage.

Overall, correlation coefficients were low, and no clear associations were identified between the analyzed variables and clinical outcomes within this exploratory dataset. These findings should be interpreted cautiously given the limited sample size and low number of outcome events.

**Figure 4:**
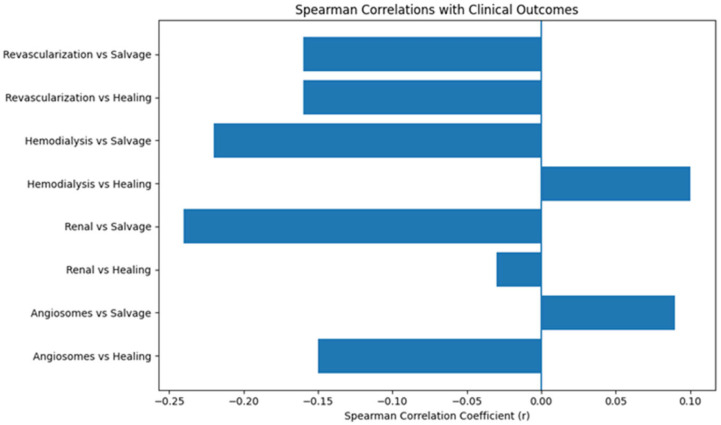
Spearman correlation coefficients with clinical outcomes.

Figure 4 illustrates these results.

Associations between patient characteristics and the outcomes of wound healing and limb salvage are presented in Table 3. Interpretations are descriptive and should not be considered evidence of statistically significant independent associations.

Considering the exploratory nature of this reinterpretation and the relatively small cohort, these findings should be interpreted with caution and should not be viewed as evidence of a validated wound-oriented perfusion model.

## 4. Discussion

### 4.1. Interpretation of Findings

Within the limitations of this exploratory dataset, no clear associations were identified between the analyzed anatomical or clinical variables and wound healing or limb salvage at 12 months. The findings suggest that anatomical classification alone may not fully explain the variability observed in clinical outcomes in patients with CLTI [8,10,12,17].

Rather, the results support the possibility that clinical evolution is influenced by multiple interacting factors, including arterial inflow, collateral circulation, microvascular integrity, local wound conditions, and overall systemic disease burden [10,11,12,17,19].

Given the limited sample size and exploratory nature of the analysis, the absence of statistically significant associations should not be interpreted as evidence that such relationships do not exist.

### 4.2. Integration with Existing Literature

The findings of the present study are in line with an increasing body of literature reporting heterogeneous outcomes associated with angiosome-guided revascularization [19,20,21]. Although several observational studies and meta-analyses have suggested a potential benefit of direct revascularization, these results are not consistently reproducible, particularly in patients with advanced disease, diffuse atherosclerosis, or multi-territorial wounds [7,9,21].

An increasing number of studies emphasize the role of collateral circulation and microvascular function in determining tissue viability and wound healing [8,10,12,17]. In patients with well-developed collateral networks, indirect revascularization may achieve outcomes comparable to direct approaches, suggesting that effective perfusion may extend beyond predefined angiosomal boundaries [8,17,20,22].

The present findings align with this perspective, as no clear advantage of direct over indirect revascularization was observed. This is consistent with the possibility that restoration of effective perfusion, rather than anatomical alignment alone, may play an important role in determining clinical outcomes [15,16].

Importantly, these results do not contradict the angiosome model but rather highlight its limitations when applied in isolation [21]. In this context, a complementary framework that incorporates both anatomical and functional aspects of perfusion may be required [15,16].

### 4.3. The Woundosome Concept

In this context, the woundosome concept may be considered a provisional and descriptive framework intended to capture the functionally relevant perfusion environment of a wound.

The concept is introduced to emphasize that wound-level perfusion may result from the combined contribution of direct arterial inflow, collateral pathways, multivessel supply, and local microvascular conditions, which are not fully captured by conventional angiosome classification [15,16,17].

Rather, it is proposed as a heuristic interpretive construct intended to provide a conceptual framework for understanding why different anatomical revascularization strategies may be associated with similar observed outcomes in certain clinical scenarios [14,20].

As such, the value of this analysis lies not in establishing a new treatment paradigm, but in highlighting the limitations of purely anatomical models and in supporting the need for a more integrated, functionally oriented approach to perfusion assessment. The woundosome concept should therefore be regarded as complementary to, rather than a replacement for, angiosome-guided revascularization strategies.

Its clinical relevance remains to be established and requires prospective evaluation using objective perfusion assessment methods [12,14,18].

Some observations from the present cohort may help place the conceptual interpretation proposed in this study into clinical context. For example, several patients classified as indirect revascularization showed favorable wound healing despite the lack of direct target-artery alignment. Although formal wound-level perfusion assessment was not available, these findings may reflect the contribution of collateral circulation, pedal arterial connections, or multivessel perfusion extending beyond conventional angiosome boundaries [8,13,16,22].

In contrast, complete wound healing was not achieved in all patients who underwent anatomically direct revascularization. This suggests that restoration of macroscopic arterial flow alone may not fully determine tissue recovery in CLTI. Other factors, including local wound characteristics, microvascular dysfunction, infection burden, and overall systemic disease severity, are also likely to influence clinical evolution [10,11,12,17,19].

These observations should not be viewed as validation of the woundosome concept, but rather as illustrative examples supporting a broader wound-oriented interpretation of perfusion heterogeneity in CLTI.

### 4.4. Clinical Implications

From a clinical perspective, the present findings do not support abandoning angiosome-guided revascularization strategies, which remain a rational and widely accepted approach. However, within the limitations of this exploratory analysis, no statistically significant differences were observed between direct and indirect revascularization in terms of wound healing or limb salvage. This observation should be interpreted with caution, as the study did not include standardized clinical assessment of wound characteristics or detailed follow-up parameters specific to wound evolution.

Therefore, these findings do not establish equivalence between revascularization strategies, but rather reflect the constraints of the available dataset. They should not be used to guide clinical decision-making in isolation. Instead, they highlight the need for a more comprehensive and clinically integrated assessment of perfusion that extends beyond anatomical target-artery alignment.

Future developments of the proposed wound-oriented perfusion concept may benefit from integration with established clinical staging systems such as WIfI, together with adjunctive physiological or microcirculatory assessment tools, including tissue oxygenation measurements, fluorescence-based perfusion imaging, or dynamic markers of wound healing. Such approaches may help better characterize the relationship between macroscopic arterial flow and effective tissue recovery.

### 4.5. Limitations

This study has several limitations that should be acknowledged.

First, this study is a retrospective reanalysis of a previously published single-center cohort with a relatively small number of patients, which limits the strength and generalizability of the findings. In addition, the number of outcome events was too low to support reliable multivariable analysis.

Another important limitation is the incomplete integration of WIfI staging into the present reanalysis. Although WIfI-related information was partially available in the original cohort and had been reported in the previous publication, the data were not sufficiently complete or consistently recorded to allow reliable incorporation into the current exploratory framework. This is particularly relevant considering the wound-oriented focus of the study, since WIfI remains one of the most widely used clinical staging systems for assessing wound severity in CLTI.

The absence of objective perfusion measurements, such as transcutaneous oxygen pressure, skin perfusion pressure, or perfusion imaging, further limits the ability to directly assess wound-level perfusion and to validate the proposed wound-oriented interpretation.

Mixed revascularization cases were excluded from inferential analyses because of their heterogeneous perfusion characteristics. Although this approach was adopted to preserve consistency within the comparative analyses, it may have introduced selection bias and limited the evaluation of more complex perfusion patterns. Alternative analytical approaches for heterogeneous perfusion profiles should be explored in larger future studies.

In addition, the use of available-case analysis due to missing data may have introduced additional bias and variability in sample size across analyses.

Finally, the woundosome concept, as applied in the present study, should be regarded as a descriptive and hypothesis-generating framework that requires prospective validation before any clinical application can be considered.

### 4.6. Future Directions

Future studies should focus on integrating objective functional perfusion assessment methods, such as transcutaneous oxygen pressure, skin perfusion pressure, or advanced imaging techniques, to better characterize wound-level perfusion and its relationship with clinical outcomes [12,17].

Prospective studies are needed to evaluate whether a wound-oriented perfusion approach, as conceptualized by the woundosome framework, can improve clinical outcomes and guide revascularization strategies more effectively than purely anatomical models [15,16].

## 5. Conclusions

The present exploratory reinterpretation does not propose a validated wound-oriented perfusion model, but instead emphasizes the limitations of relying solely on anatomical angiosome classification when interpreting clinical outcomes in CLTI. The observations from this cohort suggest that wound healing and limb salvage are probably influenced by multiple interacting factors beyond macroscopic arterial anatomy, including collateral circulation, microvascular status, local wound conditions, and overall systemic disease burden.

Within this context, the woundosome should be viewed primarily as a descriptive and hypothesis-generating conceptual framework intended to support a broader interpretation of perfusion heterogeneity, rather than as a validated clinical or physiological model. Further prospective studies incorporating objective perfusion assessment and standardized wound evaluation will be necessary before wound-oriented perfusion concepts can be meaningfully validated in clinical practice.

## Figures and Tables

**Table 1:** Main differences between the previously published angiosome-based study and the present exploratory wound-oriented reanalysis. The table summarizes the differences in study design, analytical framework, patient selection, and interpretative approach between the original cohort study and the current conceptual reinterpretation.

Aspect	Previous Study (JCM 2024) [7]	Present Study
Main objective	Evaluation of angiosome-targeted infrapopliteal angioplasty and associated clinical outcomes	Exploratory reinterpretation of perfusion patterns from a wound-oriented perspective
Study design	Retrospective observational cohort study	Secondary exploratory reanalysis of the previously published cohort
Patient cohort	Original cohort of 51 CLTI limbs	Same previously published cohort
Rutherford classification	Rutherford class 5–6 CLTI	Same cohort characteristics
WIfI data	Partially reported descriptively	Not systematically integrated into the exploratory analytical framework
Revascularization groups	Direct (*n* = 28), indirect (*n* = 12), and mixed (*n* = 11) revascularization patterns	Exploratory comparison focused on direct and indirect perfusion patterns; mixed revascularization cases (*n* = 11) were excluded from binary comparative analyses
Statistical approach	Comparative analysis of angiosome-targeted revascularization outcomes	Simplified exploratory analysis focused on perfusion-oriented interpretation
Conceptual framework	Classical angiosome concept	Exploratory “woundosome” perfusion-oriented interpretation
Main analytical focus	Anatomical angiosome-targeted revascularization	Conceptual interpretation of wound perfusion heterogeneity
Main outcomes evaluated	Wound healing, limb salvage, amputation-free survival	Reinterpretation of perfusion-outcome relationships
New patients/interventions	None	None
Nature of conclusions	Clinical outcome-oriented	Hypothesis-generating and conceptual

**Table 2:** Clinical outcomes according to revascularization strategy (available-case analysis).

Outcome	Direct Revascularization (*n* = 28)	Indirect Revascularization (*n* = 12)	*p*-Value
Wound healing at 12 months	24/28 (85.7%)	11/12 (91.7%)	1.00
Limb salvage at 12 months	24/28 (85.7%)	10/12 (83.3%)	1.00
Minor amputation	3/28 (10.7%)	1/12 (8.3%)	1.00
Major amputation	2/28 (7.1%)	1/12 (8.3%)	1.00
Mortality	3/28 (10.7%)	1/12 (8.3%)	1.00

**Table 3:** Summary of exploratory trends in selected variables and clinical outcomes.

Variable	Observed Trend with Wound Healing	Observed Trend with Limb Salvage	Interpretation
Hemodialysis	Descriptive tendency toward worse outcomes	Descriptive tendency toward worse outcomes	May reflect systemic disease burden
Renal insufficiency	No consistent association	Descriptive tendency toward worse outcomes	May reflect systemic disease burden
Creatinine	Descriptive tendency toward worse outcomes	Descriptive tendency toward worse outcomes	Marker of renal dysfunction
Number of angioplasties	Descriptive tendency toward worse outcomes	Descriptive tendency toward worse outcomes	May reflect lesion complexity
Multi-territorial wounds	Descriptive tendency toward worse outcomes	Descriptive tendency toward worse outcomes	May be associated with increased perfusion complexity
Coronary artery disease	Descriptive tendency toward worse outcomes	Descriptive tendency toward worse outcomes	May reflect systemic atherosclerotic burden
Diabetes (insulin-treated)	No consistent association	No consistent association	Limited predictive value in this cohort
Revascularization type	No consistent association	No consistent association	No clear relationship observed within this exploratory cohort

## Data Availability

The data that support the findings of this study are available from the corresponding authors upon reasonable request.

## Abbreviations

The following abbreviations are used in this manuscript:

CLTI	Chronic limb-threatening ischemia
DR	Direct revascularization
EVT	Endovascular therapy
IR	Indirect revascularization
WIfI	Wound, Ischemia, and Foot Infection
